# Human Mass Balance and Metabolite Profiling of [^14^C]‐Pamiparib, a Poly (ADP‐Ribose) Polymerase Inhibitor, in Patients With Advanced Cancer

**DOI:** 10.1002/cpdd.943

**Published:** 2021-04-19

**Authors:** Song Mu, Daniel Palmer, Richard Fitzgerald, Claudia Andreu‐Vieyra, Heather Zhang, Zhiyu Tang, Dan Su, Srikumar Sahasranaman

**Affiliations:** ^1^ BeiGene USA, Inc. San Mateo California USA; ^2^ Liverpool CR UK/NIHR Experimental Cancer Medicine Centre University of Liverpool and Clatterbridge Cancer Centre Liverpool UK; ^3^ NIHR Royal Liverpool and Broadgreen Clinical Research Facility Liverpool University Hospitals Liverpool UK; ^4^ BeiGene (Beijing) Co., Ltd. Beijing China

**Keywords:** absorption/metabolism/excretion, DNA repair, metabolite identification, pamiparib, pharmacokinetics

## Abstract

Pamiparib, a selective poly (ADP‐ribose) polymerase 1/2 inhibitor, demonstrated tolerability and antitumor activity in patients with solid tumors at 60 mg orally twice daily. This phase 1 open‐label study (NCT03991494; BGB‐290‐106) investigated the absorption, metabolism, and excretion (AME) of 60 mg [^14^C]‐pamiparib in 4 patients with solid tumors. The mass balance in excreta, blood, and plasma radioactivity and plasma pamiparib concentration were determined along with metabolite profiles in plasma, urine, and feces. Unchanged pamiparib accounted for the most plasma radioactivity (67.2% ± 10.2%). Pamiparib was rapidly absorbed with a median time to maximum plasma concentration (C_max_) of 2.00 hours (range, 1.00‐3.05 hours). After reaching C_max_, pamiparib declined in a biphasic manner, with a geometric mean terminal half‐life (t_1/2_) of 28.7 hours. Mean cumulative [^14^C]‐pamiparib excretion was 84.7% ± 3.5%. Pamiparib was mainly cleared through metabolism, primarily via N‐oxidation and oxidation of the pyrrolidine ring. A dehydrogenated oxidative product (M3) was the most abundant metabolite in biosamples. A mean of 2.11% and 1.11% of [^14^C]‐pamiparib was excreted as unchanged pamiparib in feces and urine, respectively, indicating near‐complete absorption and low renal clearance of parent drug. Cytochrome P450 (CYP) phenotyping demonstrated CYP2C8 and CYP3A involvement in pamiparib metabolism. These findings provide an understanding of pamiparib AME mechanisms and potential drug‐drug interaction liability.

The poly (ADP‐ribose) polymerase (PARP) family of proteins plays a role in DNA damage repair, DNA replication, and transcriptional regulation.^1^ PARP 1, 2, and 3 proteins recognize single‐strand breaks (SSBs) in DNA and respond by synthesizing branched poly (ADP‐ribose) chains, which recruit proteins to repair the breaks.^2^, ^3^ Inhibition of PARP enzymes allows for accumulation of unrepaired SSBs, which are converted to double‐strand breaks (DSBs) during cell division, thereby leading to genomic instability and cell death. Normal cells repair DSBs in DNA using homologous recombination (HR) pathways.^2^ Mutations in *BRCA1* and *BRCA2* are associated with deficiencies in HR repair^4^; thus, HR‐deficient cancer cells are unable to repair DNA DSBs.

Small‐molecule PARP inhibitors (PARPi) are used as therapeutic agents for malignancies harboring *BRCA1/2* mutations. Although the mechanism of action for PARPi is not yet fully elucidated, the inhibitors have been found to act by directly binding and inhibiting the enzymatic activity of PARP, thus preventing DNA repair, and by trapping PARP‐DNA complexes at the site of DNA damage.^2^ When used as a single agent in ovarian cancer patients with *BRCA1*‐ or *BRCA2*‐mutated tumors who have been treated with 2 or more lines of chemotherapy, PARPi, such as olaparib, rucaparib, and niraparib, have demonstrated sustained antitumor responses while achieving a favorable safety profile.^5^, ^6^

Pamiparib (BGB‐290) is an orally administered selective PARP1/2 inhibitor under investigation as a treatment for a variety of solid tumor malignancies. Efforts to develop fused tetra‐ or pentacyclic dihydrodiazepinoindolone derivatives with excellent PARP enzymatic and cellular PARylation inhibition activities led to the identification of pamiparib, which displays exceptional PARP1 and PARP2 inhibition, with an IC_50_ of 1.3 and 0.9 nM, respectively.^7^ In a cellular PARylation assay, pamiparib inhibited PARP activity, with an IC_50_ of 0.2 nM. Importantly, x‐ray cocrystal structural analysis of pamiparib and PARP1 showed similar binding sites with other known PARPi.^7^ In a *BRCA1* mutant mouse xenograft model, pamiparib has shown strong antitumor activity and was more potent than olaparib.^8^ Pamiparib has also demonstrated brain penetrance after oral administration in mice.^9^ Results from a first‐in‐human dose‐escalation/dose‐expansion study (BGB‐290‐AU‐002; NCT02361723) demonstrated that pamiparib was generally well tolerated up to 60 mg with a linear pharmacokinetic (PK) profile across the tested dose range (2.5‐120 mg) and promising antitumor activity.^10^ This first‐in‐human study also established the recommended phase 2 dose (RP2D) of pamiparib as 60 mg orally twice daily.^10^ The preliminary antitumor activity, safety/tolerability profile, and RP2D of pamiparib have been confirmed in a phase 1/2 open‐label multicenter study in Chinese patients with advanced high‐grade ovarian cancer (BGB‐290‐102; NCT03333915).^11^ Development of investigational agents and their subsequent clinical use in different special populations relies on a clear understanding of their disposition in patients. As such, the goal of the current study (BGB‐290‐106; NCT03991494) was to assess the absorption, metabolic pathways, and excretion routes of pamiparib in patients with advanced solid tumors. Safety in these patients was also evaluated. Consistent with its mechanism of action, pamiparib has demonstrated clastogenic activity in an in vitro chromosomal aberration assay using mammalian Chinese hamster ovary cells and an in vivo rat bone marrow micronucleus assay (data on file). Consequently, pamiparib cannot be given to healthy volunteers and was administered to patients with cancer in this study. The results from this study will be used to guide future study designs evaluating the potential for drug‐drug interactions.

## Methods

### Study Design and Patient Population

This was a phase 1 open‐label, single‐center inpatient study investigating the absorption, metabolism, and excretion (AME) of pamiparib in patients with advanced and/or metastatic solid tumors who progressed from standard therapy or for whom no standard therapy existed. Adult patients (≥18 years of age) with histologically or cytologically confirmed locally advanced or metastatic solid tumors with Eastern Cooperative Oncology Group performance status ≤ 1 and adequate organ function were eligible. Patients were excluded from the study if they had clinically significant cardiovascular disease; previous complete gastric resection, chronic diarrhea, active inflammatory gastrointestinal disease, or any other disease causing malabsorption syndrome; poor peripheral venous access; major surgical procedure, open biopsy, or significant traumatic injury ≤ 2 weeks prior to day 1, or anticipation of need for major surgical procedures during the study; or had participated in a clinical trial involving a radiolabeled investigational product < 12 months prior to check‐in.

Part 1 of the study included a screening phase of up to 28 days prior to drug administration to assess eligibility. Patients were admitted to the clinical research unit (CRU) at Royal Liverpool University Hospital (Liverpool, UK)/Clatterbridge Cancer Centre NHS Foundation Trust (Bebington, Wirral, UK) on day −1 and received a single dose of 60‐mg/100 μCi [^14^C]‐pamiparib orally the morning of day 1. Patients remained in the CRU until completion of assessments on day 7, when discharge criteria of ≈90% of the radioactive dose being recovered or <1% of the radioactive dose being recovered in urine and feces for 2 consecutive 24‐hour collection intervals were met. If discharge criteria were not met by day 7, patients collected 24‐hour excreta samples up to day 14 on an outpatient basis. Part 2 of the study, evaluating the safety/tolerability and antitumor activity of 60 mg twice‐daily pamiparib in patients with advanced and/or metastatic solid tumors, is ongoing and is not included in this report.

The study was conducted in accordance with Good Clinical Practice and all applicable regulatory requirements, including the Declaration of Helsinki. All patients provided written informed consent. Prior to the start of the study, the protocol, protocol amendments, investigator's brochure, and informed consent form were reviewed and approved by the local ethics committee (North West‐Greater Manchester Central Research Ethics Committee; Manchester, UK) and the Administration of Radioactive Substances Advisory Committee.

### Study Drug and Materials

[^14^C]‐Pamiparib was supplied by Arcinova (Alnwick, UK) as powder in vials. The investigational product was reconstituted into a suspension by adding water and mixed thoroughly at Covance CRU (Leeds, UK). The formulation was transported to the CRUs the night before dosing. Chemical stability and radiopurity of the suspension were established for 48 hours at room temperature (data on file). The total radioactive dose administered to each patient was calculated from the weight of test material per vial administered and the specific activity of the test material after correcting for the amount of residual radioactivity in the dose washes. Unlabeled pamiparib (C_16_H_15_FN_4_O), BGB‐326 (C_16_H_16_N_4_O, internal standard), and reference standards for metabolites M1 (BGB‐4033, C_16_H_15_FN_4_O_2_) and M2 (BGB‐13257, C_16_H_15_FN_4_O_2_) were provided by BeiGene, Ltd. Other analytical‐grade chemicals and solvents were obtained from commercial sources.

### Drug Administration

Patients fasted overnight and 2 hours after oral administration of a single 60‐mg dose containing ≈100 μCi (3.7‐3.9 MBq) of [^14^C]‐pamiparib with ≈240 mL of room‐temperature water or juice (orange or grape). The residual radioactivity present in each vial postdose was corrected from the dose. The radioactive dose of 100 μCi of [^14^C]‐pamiparib provided minimum risk to patients according to the dosimetry calculation from a quantitative whole‐body autoradiography study in male and female Long Evans rats; the dose also provided sufficient signal for total radioactivity counting and quantitative radioprofiling in blood, plasma, and excreta (data on file). Patients were not permitted to lie in the supine position for 2 hours after drug administration, except if required because of an adverse event (AE) and/or study procedure.

Supportive therapy (eg, analgesics, anticonvulsants, anti‐emetics, antidiarrheals, laxatives, opiates, and transfusion of blood products) considered necessary for patient welfare was provided at the discretion of the investigator. Hormone replacement therapy was allowed, as was the use of bisphosphonates and denosumab if a patient was receiving a stable dose > 28 days before enrollment. Other anticancer therapy (surgery; chemotherapy; radiation therapy; immunotherapy; investigational agents; cytotoxic, biologic, or hormone therapy; anticancer Chinese medicine; or herbal remedies ≤ 14 days or ≤ 5 half‐lives [whichever was shorter]) was not permitted prior to the first dose and during the study. Strong/moderate cytochrome P450 (CYP) 3A inhibitors or strong CYP3A inducers were not permitted ≤ 10 days or ≤ 5 half‐lives (whichever was shorter) prior to day 1 and during the study.

### Sample Preparation

Total radioactivity was analyzed at Covance Laboratories (Harrogate, North Yorkshire, UK), and metabolite profiling was conducted at Covance Laboratories (Madison, Wisconsin). Determination of pamiparib concentrations in plasma and total radioactivity in plasma and whole blood was performed predose and 0.25, 0.5, 1, 1.5, 2, 2.5, 3, 6, 12, 24, 48, 72, 96, 120, and 144 hours postdose. Pamiparib concentrations in plasma and urine were determined at Covance Laboratories (Salt Lake City, Utah) with validated liquid chromatography‐tandem mass spectrometry (LC‐MS/MS) methods.

Total radioactivity concentrations of [^14^C]‐pamiparib in whole blood, plasma, urine, and feces were determined by liquid scintillation counting (LSC). Radioactivity in each sample was measured for 5 minutes using a Packard Tri‐Carb liquid scintillation counter model 2100TR, 2900TR, or 3100TR (Canberra Packard, Pangbourne, Berks, Australia) with the facilities for computing quench‐corrected disintegrations per minute. Metabolite identification in plasma, urine, and feces was conducted by LC‐MS. Quantitation of the metabolites present in plasma, urine, and feces was based on the profiles of radioactivity. The limit of quantitation for radioactivity in plasma, urine, and feces was set at 1% of each chromatographic analysis (run) and 10 counts per minute (cpm) peak height; therefore, radioactive peaks that were <1% of the total run or <10 cpm peak height were reported as not detected. In addition, pamiparib and standards were reported, regardless of the percentage of run or peak height.

#### Plasma and Whole Blood

Each aliquot of plasma (about 800 μL) was added directly to liquid scintillant prior to LSC. Whole blood (about 400 μL) was subjected to combustion analysis prior to LSC. Samples were combusted using a Packard Sample Oxidiser model 307. The combusted products were absorbed in Carbosorb (PerkinElmer LAS [UK] Ltd, Beaconsfield, Buckinghamshire, UK) and mixed with Permafluor E+ scintillation fluid (PerkinElmer LAS [UK] Ltd).

For metabolite profiling in plasma, ≈1.9 to 2.8 g of each plasma sample collected 0.5, 1, 2, 6, 12, and 24 hours postdose was extracted twice with 6 mL of 0.1% formic acid in acetonitrile (ACN). Duplicate aliquots were analyzed by LSC to determine extraction recoveries, which ranged from 77.4% to 98.5%. The combined supernatants were evaporated to dryness under nitrogen and reconstituted in 2:1 reverse osmosis water:methanol (MeOH). Duplicate aliquots were analyzed by LSC to determine reconstitution recoveries, which ranged from 89.3% to 115%. The reconstituted samples were analyzed by LC‐MS, with eluent fractions collected at 10‐second intervals into 96‐well plates containing solid scintillant. Radioactivity in each well was determined using TopCount analysis, and radiochemical profiles were generated based on radioactivity counts.

#### Urine

Each aliquot of urine (about 1 mL, weighed) was added directly to liquid scintillant prior to LSC. For metabolite profiling, urine samples collected 0‐4, 4‐8, 8‐12, 12‐24, 24‐48, 48‐72, and 72‐96 hours postdose were pooled by individual patient to generate 0‐ to 96‐hour pooled samples including 0.2% of each sample by weight. The radioactivity in each pooled sample was determined by LSC. The total amount of urine collected was factored into the recovery calculation. A 1‐mL subsample of each pooled urine sample was centrifuged, and duplicate aliquots were analyzed by LSC to determine the recovery of radioactivity, which ranged from 95.8% to 102%. Pooled urine samples were analyzed by LC‐MS, with eluent fractions collected at 10‐second intervals into 96‐well plates containing solid scintillant. Radioactivity in each well was determined using TopCount analysis, and radiochemical profiles were generated based on radioactivity counts.

#### Feces

Portions of fecal homogenates (about 200‐500 mg) were subjected to combustion analysis prior to LSC. For metabolite profiling, feces samples collected 0‐24, 24‐48, 48‐72, 72‐96, and 96‐120 hours postdose were pooled by individual patient to generate 0‐ to 120‐hour pooled samples including 0.5% of each sample by weight. Feces were pooled per patient (if required) according to the appropriate 24‐hour collection period. The samples were homogenized in deionized water, avoiding excessive dilution (data on file). The radioactivity in each pooled sample was determined by LSC. The total amount of feces collected was factored into the recovery calculation. Approximately 1.6 to 2.7 g of each pooled feces sample was extracted twice with 6 mL of 0.1% formic acid in ACN. Duplicate aliquots were analyzed by LSC to determine extraction recoveries, which ranged from 76.6% to 89.7%. The combined supernatants were evaporated to dryness under nitrogen and reconstituted in 300 μL of MeOH. Samples were sonicated, vortex‐mixed, and centrifuged, and duplicate aliquots were analyzed by LSC to determine reconstitution recoveries, which ranged from 96.5% to 102%. The reconstituted samples were analyzed by LC‐MS, with eluent fractions collected, and radiochemical profiles were generated based on radioactivity counts.

### Bioanalytical Procedures

#### Validated LC‐MS/MS Methods for Pamiparib in Plasma and Urine

An aliquot of human plasma (25 μL) containing dipotassium ethylenediaminetetraacetic acid as anticoagulant or urine (25 μL) was added with the internal standard (20.0 μL of 400 ng/mL BGB‐326) in water:ACN (50:50 v/v) followed by the addition of water:ACN (50:50 v/v, 20.0 μL). The plasma samples were centrifugated and vortexed followed by the addition of 200 μL of 5% NH_4_OH and 100 μL of water. After centrifugation, the supernatant (50 μL) was loaded to Biotage Isolute supported liquid extraction plate (Biotage, Holliston, Massachusetts) and eluted with methyl tert‐butyl ether (700 μL). The eluted samples were dried down at 45°C in TurboVap for 30 minutes and reconstituted in 400 μL of water:MeOH (80:20 v/v), followed by LC‐MS/MS analysis. The urine samples were centrifuged and vortexed followed by the addition of 200 μL of ACN. After centrifugation, 100 μL of the samples was transferred to a plate followed by the addition of 300 μL of water. The samples were centrifugated again before LC‐MS/MS analysis. The LC‐MS/MS instrumentation was the same for plasma and urine analysis including a LEAP autosampler (LEAP Technologies, Chapel Hill, North Carolina), a Shimadzu SCL‐10A controller with LC‐10AD pump (Shimadzu, Columbia, Maryland), and a Sciex API 5000 mass spectrometer (Sciex, Framingham, Massachusetts) equipped with a XBridge Phenyl column (2.1 × 50 mm, 5 μm; Waters, Pleasanton, California). The mobile phase A is 10 mM ammonium formate in water, and mobile phase B is MeOH:ACN (50:50 v/v). Chromatographic separation was performed at a flow rate of 0.3 mL/min using a gradient elution program of 3.5 minutes, starting from 25% of mobile phase B/75% A for 0.5 minutes, gradually changing to 75% B in 2 minutes, holding for 0.5 minutes, and back over to 25% B within 0.1 minutes. The calibration range was 1.00 to 1000 ng/mL for both plasma and urine analyses. Linear regression with weighting factor 1/χ^2^ was used to generate the standard curves.

#### Metabolite Profiling

Metabolic profiling was performed on plasma, urine, and feces samples using LC‐MS. Shimadzu/Nexera LC‐30AD pumps and Shimadzu/Prominence DGU‐20A5R degasser were used with Shimadzu/Prominence controller CBM‐20A. The high‐pressure liquid chromatograph (HPLC) was equipped with an autoinjector (Shimadzu/Nexera SIL‐30ACMP) set at 10°C and a fraction collector (Leap Technologies PAL HTC‐xt) set at 15°C. Thermo Fisher Scientific Q Exactive was the mass spectrometer system. The LC‐MS condition is displayed in Table 1.

**Table 1:** LC‐MS Conditions

Ionization interface	Positive electrospray interface		
HPLC column	Waters XBridge C18, 4.6 × 250 mm, 3.5 μm (oven temperature, 30°C)		
Mobile phase A	0.1% formic acid in reverse osmosis water		
Mobile phase B	0.1% formic acid in acetonitrile		
Gradient	Time (minutes)	% A	% B
	Initial	95	5
	5.00	95	5
	45.00	80	20
	55.00	45	55
	56.10	5	95
	61.00	5	95
	61.10	95	5
	66.00	95	5
Flow rate	1.00 mL/min; split ratio, 80:20; mass spectrometer: fraction collector		
Survey scan	*m/z* 150‐800 at 70 000 resolution		
Dependent scans	MS^2^ at 17 500 resolution		
S‐Lens RF level	50.00		
Source voltage	3.5 Kv		
Capillary temperature	320°C		
Source temperature (probe heater)	320°C		

HPLC, high‐performance liquid chromatography; LC‐MS, liquid chromatography‐mass spectrometry; RF, radio frequency.

### PK Parameters

Standard PK parameters of pamiparib in plasma as well as total radioactivity in blood and plasma were calculated with noncompartmental methods with Phoenix WinNonlin (version 8.1). Area under the concentration‐time curve (AUC) from time 0 to infinity (AUC_0‐∞_), AUC from time 0 to last quantifiable concentration (AUC_0‐t_), C_max_, time postdose at C_max_ (t_max_), t_1/2_, and apparent total clearance (CL/F) were calculated. Renal clearance (CL_R_) was calculated as Ae/AUC, where Ae was the amount of drug excreted in urine.

### Safety Evaluations

Safety and tolerability were evaluated throughout the study, and AEs were assessed from the time of obtaining informed consent to discharge from the study and follow‐up visit or early withdrawal. Scheduled safety assessments of 12‐lead electrocardiogram, physical examination, vital signs, and clinical laboratory investigations (clinical chemistry, hematology, serology, and urinalysis) occurred before administration of the study drug and at discharge and follow‐up visit.

### Metabolic Stability and Cytochrome P450 Phenotyping

Metabolic stability in human liver microsomes (HLMs) and cytochrome P450 (CYP450) phenotyping assays were conducted in 3D BioOptima (Suzhou, China) using validated methodology^12^, ^13^ to determine the intrinsic clearance and enzymes responsible for metabolism of pamiparib. HLMs for microsome stability was from BD Biosciences (Woburn, New York). For phenotyping, HLMs and recombinant P450 (rCYP) were from Corning (Woburn, New York). Pamiparib (1 μM) was incubated with pooled HLM (0.5 mg/mL), NADPH (1 mM), and MgCl_2_ (3 mM) terminated at 0, 5, 15, 30, 60, and 90 minutes. The intrinsic clearance was calculated as 0.693/(t_1/2_ × HLM concentration), where t_1/2_ is the half‐life of pamiparib determined in HLMs. For phenotyping in HLMs, pamiparib (1 μM) was incubated in the absence or presence of isoform‐selective inhibitors: 0.2 μM α‐naphthoflavone for CYP1A2, 2 μM clopidogrel for CYP2B6, 16 μM montelukast for CYP2C8, 10 μM sulfaphenazole for CYP2C9, 30 μM nootkatone for CYP2C19, 2 μM quinidine for CYP2D6, and 1 μM ketoconazole for CYP3A. The following isoform‐selective probe substrates served as positive controls: 40 μM phenacetin for CYP1A2, 100 μM bupropion for CYP2B6, 2 μM amodiaquine for CYP2C8, 5 μM diclofenac for CYP2C9, 40 μM S‐mephenytoin for CYP2C19, 5 μM dextromethorphan for CYP2D6, and 2 μM midazolam for CYP3A4. The incubation was maintained 90 minutes for pamiparib and 15 minutes for positive controls under the same conditions for intrinsic clearance. Pamiparib (1 μM) was also incubated with rCYP1A2, rCYP2B6, rCYP2C8, rCYP2C9, rCYP2C19, rCYP2D6, or rCYP3A4 (50 pmol/mL) for 90 minutes with NADPH and MgCl_2_. The following isoform‐selective probe substrates served as positive controls: 1 μM phenacetin for CYP1A2, 2 μM bupropion for CYP2B6, 2 μM amodiaquine for CYP2C8, 2 μM diclofenac for CYP2C9, 10 μM S‐mephenytoin for CYP2C19, 1 μM dextromethorphan for CYP2D6, and 20 μM midazolam for CYP3A4. Pamiparib, M1, and M2 were analyzed by LC‐MS/MS.

## Results

### Patients

A total of 4 PARPi therapy‐naive patients were enrolled and completed the study. Three patients were male, and all patients had metastatic disease. Patients had a diagnosis of pancreatic cancer (n = 3) or prostate cancer (n = 1) and had received 1‐2 (n = 2) or 3 or more prior (n = 2) lines of anticancer therapies. The demographic and baseline characteristics of the patients are summarized in Table 2. Excreta were collected for up to 192 hours postdose from 4 patients who remained within the CRU until day 7 (144 hours postdose). Patient 103 met the exit criteria by 144 hours. Additional collection of excreta was performed at home for patients 101, 102, and 104. Patients 101 and 102 met the exit criteria at 168 hours, whereas patient 104 met the criteria at 192 hours.

**Table 2:** Patient Demographics and Baseline Characteristics

	Patients (n = 4)
Age (years), median (range)	54.5 (52‐64)
Sex, n (%)	
Male	3 (75)
Female	1 (25)
Race, n (%)	
White	4 (100)
BMI (kg/m^2^), mean (SD)	27.6 (2.0)
ECOG performance status, n (%)	
0	0
1	4 (100)
Primary tumor location, n (%)	
Pancreas	3 (75)
Prostate	1 (25)
Site of metastatic lesion, n (%)	
Liver	3 (75)
Bone	1 (25)
Prior cancer surgery/biopsy, n (%)	4 (100)
Prior radiotherapy, n (%)	1 (25)
Prior systemic therapy, n (%)	4 (100)
Prior lines of systemic therapy, n (%)	
1	1 (25)
2	1 (25)
≥3	2 (50)

BMI, body mass index; ECOG, Eastern Cooperative Oncology Group; SD, standard deviation.

### Single‐Dose Pamiparib PK Profile

**Figure 1:**
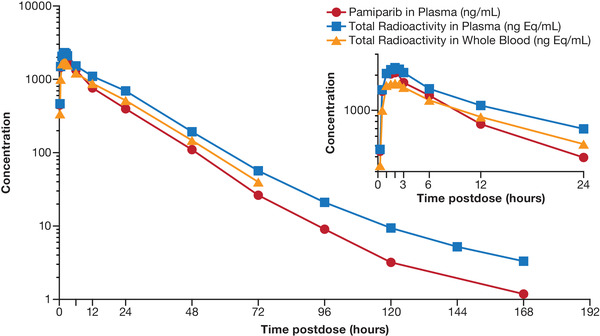
Concentration‐time curves of pamiparib plasma concentrations and plasma and blood total radioactivity after a single dose of pamiparib. Data points represent arithmetic mean. Pamiparib plasma concentrations were analyzed by liquid chromatography‐tandem mass spectrometry.

Single‐dose plasma PK parameters (eg, AUC, C_max_, t_max_) of [^14^C]‐pamiparib determined by LC‐MS/MS and total radioactivity in plasma and whole blood (Table 3) demonstrated pamiparib is rapidly absorbed, with a median t_max_ of 2.00 hours (range, 1.00‐3.05 hour). After reaching C_max_, pamiparib plasma concentrations appeared to decline in a biphasic manner (Figure 1), with a geometric mean (coefficient of variation) of t_1/2_ of 28.7 hours (22%) and an apparent clearance (CL/F) of ≈2.2 L/h (23.2%). A median t_max_ of 2.25 hours postdose was observed for both plasma and whole blood total radioactivity. In addition, levels of total radioactivity in plasma appeared to decline in a biphasic manner, with a geometric mean t_1/2_ of 19.6 hours; in whole blood, levels of total radioactivity declined in a biphasic manner, with a geometric mean t_1/2_ of 13.0 hours.

**Table 3:** Plasma Pharmacokinetic Parameters After a Single Dose of [^14^C]‐Pamiparib (60 mg, 100 μCi) to Patients

	Plasma Pamiparib^a^ (n = 4)	Plasma Total Radioactivity (n = 4)	Whole Blood Total Radioactivity (n = 4)
AUC_0–∞_, ng·h/mL or ngEq·h/g			
Arithmetic mean (SD)	29 700 (5780)	42 900 (8380)	32 500 (4860)
Geometric mean (CV%)	29 300 (21.6)	42 200 (21.5)	32 200 (16.2)
AUC_0–t_, ng·h/mL or ngEq·h/g			
Arithmetic mean (SD)	29 700 (5760)	42 500 (8310)	31 500 (4650)
Geometric mean (CV%)	29 200 (21.6)	41 800 (21.6)	31 200 (16.1)
C_max_, ng/mL or ngEq/g			
Arithmetic mean (SD)	2210 (277)	2400 (215)	1740 (212)
Geometric mean (CV%)	2190 (12.4)	2390 (9.0)	1730 (11.8)
t_max_, h	2.00 (1.00, 3.05)	2.25 (1.50, 3.05)	2.25 (1.50, 3.05)
t_1/2_, h			
Arithmetic mean (SD)	29.2 (6.02)	21.6 (10.3)	13.3 (3.13)
Geometric mean (CV%)	28.7 (22.0)	19.6 (55.9)	13.0 (24.1)
CL/F, L/h			
Arithmetic mean (SD)	2.25 (0.55)	―	―
Geometric mean (CV%)	2.21 (23.2)	―	―
AUC_0‐∞_ ratio			
Blood/plasma			
Arithmetic mean (SD)	―	―	0.765 (0.044)
Geometric mean (CV%)	―	―	0.764 (5.8)
Plasma pamiparib/total radioactivity			
Arithmetic mean (SD)	―	0.694 (0.185)	―
Geometric mean (CV%)	―	0.694 (2.7)	―

AUC_0‐∞_, area under the concentration‐time curve from time 0 to infinity; AUC_0–t_, area under the concentration‐time curve from time 0 to last quantifiable time; CL/F, apparent clearance; C_max_, maximum observed plasma concentration; CV%, percent coefficient of variation; SD, standard deviation; t_1/2_, elimination half‐life; t_max_, time to reach C_max_.

Median (min, max) is presented for t_max_.

^a^ Based on liquid chromatography‐tandem mass spectrometry analysis.

LC‐MS/MS analysis of urine using a validated LC‐MS/MS method showed that the geometric mean (CV%) cumulative percent of pamiparib excreted unchanged in urine was ≈1.7% (39.6%) of the administered dose over the 192‐hour study. The geometric mean (CV%) of renal clearance was estimated as 37.4 mL/h (59.5%).

The mean total plasma radioactivity‐time profile closely followed that of the plasma pamiparib mean concentration‐time profile, with the respective geometric means for C_max_, AUC_0‐t_, and AUC_0‐∞_ of pamiparib measured by LC‐MS/MS being ≈91.6%, 69.9%, and 69.4%, respectively, of those for total radioactivity in plasma. Furthermore, the mean whole blood‐to‐plasma ratio for total radioactivity was ≈0.764 for AUC_0‐∞_, and mean concentrations of radioactivity in whole blood were consistently lower than those in plasma over the entire study's duration. This resulted in total radioactivity blood:plasma ratios < 1 (range, 0.66‐0.80) from 0.25 to 72 hours postdose.

### Excretion of Pamiparib Radioactivity

**Figure 2:**
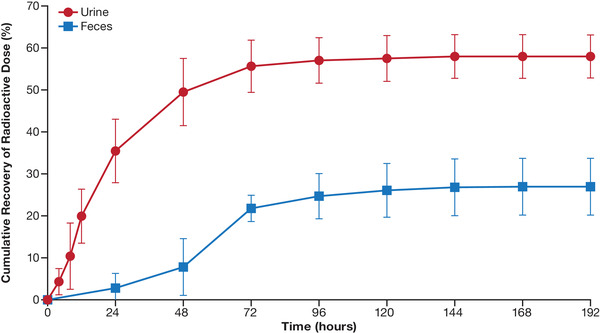
Cumulative percent of recovered radioactivity in urine and feces. Data points represent arithmetic mean (SD).

The overall mean total recovery of radioactivity in urine and feces was 84.7% ± 3.5% (range, 81.3%‐89.4%) over the 192‐hour collection period. The cumulative percent of recovered radioactivity is presented in Figure 2. The majority of the excreted dose was recovered within 72 hours in urine and within 96 hours in feces. The main route of excretion of pamiparib‐related substance was renal (mean recovery, 57.8% ± 5.1%); 26.9% ± 6.8% of the administered radioactivity was excreted in the feces. Beyond these times, a mean recovery of ≈2.4% of the dose was collected in urine over 72 to 192 hours, whereas the mean recovery in feces from 96 to 192 hours was 2.5% of the dose.

### Metabolite Profiling

**Figure 3:**
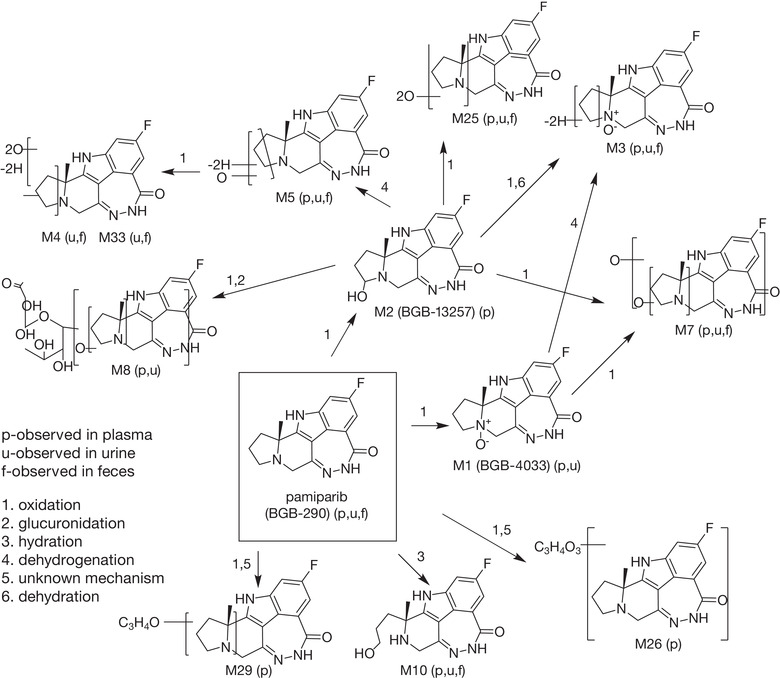
Proposed biotransformation pathways of pamiparib.

**Figure 4:**
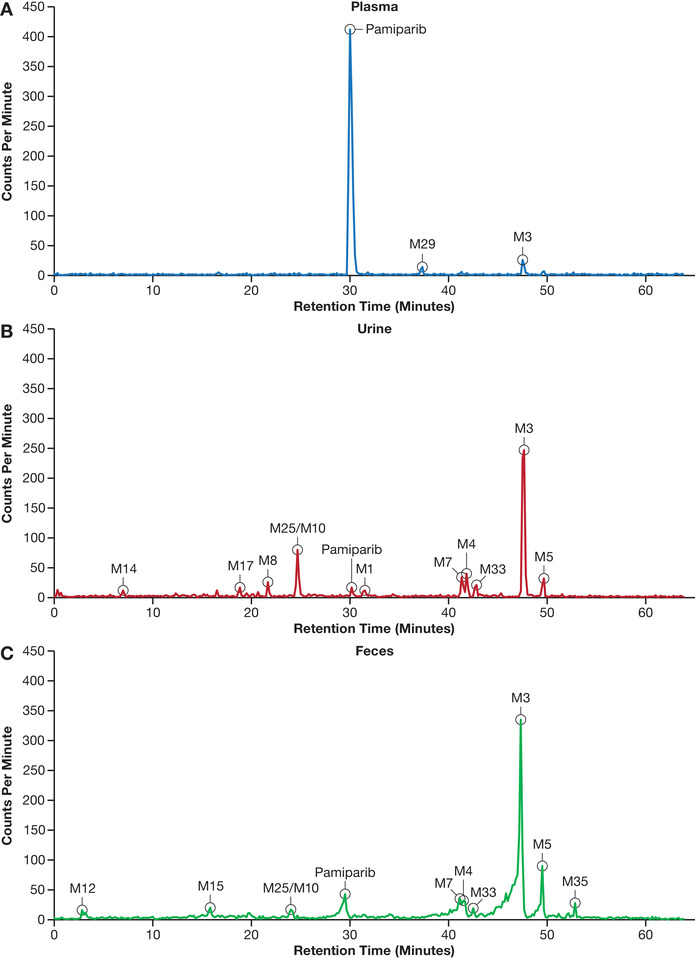
Representative radiochromatograms from 1 patient for metabolite structures eluted in (A) plasma, (B) urine, and (C) feces.

The proposed metabolic profile of [^14^C]‐pamiparib involves oxidation and dehydrogenation to produce mono‐oxidized (M1, M2, M3, M5) and dioxidized (M4, M7, M25, M33) metabolites, oxy‐glucuronidation (M8), hydration (M10), and the addition of C_3_H_4_O to pamiparib (M26) and C_3_H_4_O_3_ to pamiparib (M29); see Figure 3. In human plasma, the radioactivity of the main metabolites was quantified, and AUC_0‐24h_ values were compared with those of total radioactivity. Table 4 lists the ratios of pamiparib metabolites to total radioactivity based on AUC_0‐24h_ in plasma samples; representative radiochromatograms of metabolite structures used for analysis are presented in Figure 4. Unchanged pamiparib accounted for a mean of 67.2% (range, 55%‐78%) of total radioactivity exposure. Metabolite M3 represented 7.8% of total radioactivity exposure (6.1%‐9.5%) in human plasma.

**Table 4:** [^14^C]‐Pamiparib Metabolite to Total Radioactivity Ratios Based on AUC_0‐24h_ in Plasma Samples After a Single Oral Dose of [^14^C]‐Pamiparib to Patients (60 mg, 100 μCi)

Component Designation	Patient	Percent of Total Radioactivity Concentration
M19	S104	0.432
M2 (BGB‐13257)	S103	2.00
	S104	4.38
Pamiparib (BGB‐290)	S101	78.0
	S102	72.6
	S103	63.2
	S104	55.0
M26	S103	5.69
	S104	8.11
M3	S101	9.50
	S102	9.00
	S103	6.67
	S104	6.09

The percentages of the radioactive dose of the metabolites in urine and feces are presented in Table S1. In urine, among 52.5% of the dose quantified, 49.1% of the dose was identified as metabolites with tentative structures, 1.2% of the dose as unknown metabolites, and 2.11% as unchanged parent. The main peak observed for all patients was M3, which represented ≈50% of the HPLC run and a mean of 28.5% of the administered dose. Other minor metabolites observed were M8, M25/M10 (eluting as 1 peak on LC‐MS), M1, M7, M4, M5, M17, and M33, and all represented a mean of between 0.530% and 8.05% of the administered dose. In feces, among 17.5% of the dose quantified, 13.6% of the dose was identified as metabolites with tentative structures, 2.8% of the dose as unknown metabolites, and 1.11% as unchanged pamiparib. Six metabolites were identified among 22 radioactivity peaks. M3 was the main metabolite in feces, representing up to 51.5% of the HPLC run and 8.71% of the dose administered. M25/M10, M7, M4, and M5 represented a mean of 1.30%, 1.24%, 0.90%, and 1.32%, respectively, of the dose administered.

### Safety

Single doses of 60 mg [^14^C]‐pamiparib were considered well tolerated by male and female patients with advanced and/or metastatic solid tumors in this study. There were no deaths or serious AEs, and none of the patients were discontinued from the study because of treatment‐emergent AEs (TEAEs). Overall, 16 TEAEs were reported in 4 patients (100%). All TEAEs were mild (CTCAE grade 1), and the majority of all TEAEs were considered by the investigator to be unrelated to [^14^C]‐pamiparib (11 events [68.8%] in 4 patients). Five TEAEs (31.3%) in 2 patients were considered related to [^14^C]‐pamiparib. Pamiparib‐related TEAEs included dysgeusia (n = 2), thrombocytopenia (n = 1), blood alkaline phosphatase increase (n = 1), and gamma‐glutamyl transferase increase (n = 1).

### HLM Stability and P450 Phenotyping

Pamiparib metabolism in HLM was slow, and the half‐life and intrinsic clearance were determined to be 180 minutes and 7.90 μL·min/mg protein, respectively. During pamiparib CYP450 phenotyping in HLMs, 45.0% of pamiparib remained after a 90‐minute incubation without CYP inhibitors. The addition of CYP2C8 inhibitor (montelukast) and CYP3A4 inhibitor (ketoconazole) to the HLM incubation resulted in 82.9% and 63.1%, respectively, of pamiparib remaining at the end of the incubation period; all other CYP inhibitors had nearly no effect on pamiparib metabolism. Metabolite M1 was detected after incubation of pamiparib with rCYP3A4 and rCYP2D6, but not detected with other rCYP isoforms. Metabolite M2 was detected in all isoforms except rCYP2C9, with higher levels observed from rCYP2C8, rCYP2D6, and rCYP3A4 incubations.

## Discussion

Four adult patients with advanced and/or metastatic solid tumors were enrolled in this study to investigate the AME of [^14^C]‐pamiparib and to characterize metabolites present in plasma, urine, and feces following a single oral administration. After administration of a single 60‐mg oral dose of [^14^C]‐pamiparib to patients, pamiparib was rapidly absorbed, with a median t_max_ of 2.00 hours. The LC‐MS/MS analysis showed that the geometric means of C_max_ and AUC_0‐t_ were estimated as 2190 and 29 200 ng/mL, respectively. Those numbers were comparable to the geometric means of C_max_ of 1737 ngEq/mL and AUC_0‐t_ of 19670 ngEq·h/mL based on high‐performance liquid chromatography‐liquid solid chromatography. In addition, the exposure of pamiparib in this study was comparable to that in the first‐in‐human study following a 60‐mg single dose (BGB‐290‐AU‐002; NCT02361723; geometric mean C_max_, 1881 ng/mL; AUC_0‐inf_, 32 005 ng·h/mL; n = 11). The geometric mean for CL/F estimated from AUC_0‐∞_ was 2.21 L/h, which is low compared with human hepatic blood flow (97 L/h),^14^ consistent with its stable metabolic profile and low intrinsic clearance in human liver microsomes. Although the CL/F was comparable to that observed in the phase 1 dose‐escalation study, the geometric mean for t_1/2_ of 28.7 hours in this study was longer than the estimated t_1/2_ of 13 hours in the first‐in‐human study (BGB‐290‐AU‐002; NCT02361723). This could be because of the longer sampling scheme employed in this study, in which the PK profile was followed for up to 144 hours in this study compared with 48 hours in the first‐in‐human study.^10^ The similar CL/F estimate between the 2 studies suggests a minimum contribution of exposure from 48 hours and beyond to overall AUC. The geometric coefficient of variation of parent AUCs among 4 subjects was <22%, indicating low interindividual variability for exposure in this study.

Unchanged pamiparib was the major component in plasma. The similarity in the total plasma radioactivity‐time and plasma pamiparib concentration‐time profiles suggests that metabolites play only a minor role in contributing toward the circulating total radioactivity in plasma. The comparable t_1/2_ of pamiparib to that of total radioactivity in plasma suggested an absence of persistent metabolites. The mean AUC_0‐∞_ whole‐blood/plasma ratios for total radioactivity between 0.25 and 72 hours was ≈0.76 and was constantly < 1 (mean range, 0.66‐0.80) indicating a low association of radioactivity with red blood cells and no preferential binding or uptake of drug‐related radioactivity into blood cells over time.

Following a single oral dose of [^14^C]‐pamiparib to human patients, a mass balance higher than 80% was achieved for all 4 subjects, and interindividual variability of urine and feces recovery data was low, as the overall mean total recovery of radioactivity in excreta (urine and feces) was 84.7% ± 3.5% over the 192‐hour collection period. Renal excretion represented the primary route of elimination, accounting for a mean recovery of 57.8% ± 5.1% of the dose. Pamiparib and its metabolites had fecal excretion of 26.9% ± 6.8% of the dose.

Pamiparib underwent extensive metabolism in humans. Twelve metabolites were identified in human plasma, urine, and feces samples. The major metabolic pathways involved oxidation, with a minor contribution from phase II conjugation of oxidative metabolites and hydration. M1 and M2 were significant metabolites identified in liver microsomes of multiple species (data on file), and their authentic standards were synthesized. M1, a minor metabolite in urine (0.53% of dose), was confirmed as an N‐oxide by comparing with the standard, BGB‐4033. Although M2 was not identified in urine or feces, a significant amount of M2 was identified in the plasma of 2 subjects, but the level was below the limit of quantification in the other 2 subjects. M2 was confirmed to contain hydroxy‐pyrolidine by comparison with authentic standard BGB‐13257; however, this metabolite is not stable and easily dehydrates to give dehydrogenated product of pamiparib in the LC‐MS source or under other conditions. M3 (oxy‐dehydro‐pamiparib) is the most abundant metabolite in human plasma and excreta. It may be derived from oxidation of M1 or M2 followed by dehydration. An analogue of M3 with the same molecular weight was identified as M5 in urine (1.97% of dose) and feces (1.32% of dose). Sequential oxidation of M2 could produce dioxidized metabolites M25 (recovery unknown because of coelution with M10) and M7 (3.1% of dose in urine and 1.24% of dose in feces). Further oxidation of M5 produced a dioxidized dehydrogenated metabolite, M4, in urine (3.75% of dose) and feces (0.9% of dose). Glucuronidation of M2 resulted in generation of M8 in urine (2.15% of dose). M10 was identified as a pyrrolidine ring‐opened metabolite in urine (M25 + M10 8.05% of dose) and feces (M25 + M10 1.3% of dose) and coeluted with M25. N‐oxidation and oxidation of pyrrolidine were demonstrated to be the major oxidation pathways. No direct glucuronide of pamiparib was identified.

In human plasma, the main component was unchanged pamiparib, which represented a mean of 67% of total radioactivity concentration based on AUC_0‐24h_; the M3 metabolite was the most abundant metabolite and was observed in plasma for all patients. Metabolite M29 was observed in plasma for 2 patients, and M2 and M26 were observed in plasma of the other 2 patients. The high‐resolution LC‐MS confirmed the molecular weight and composition of 2 minor metabolites, M29 and M26, but the exact mechanisms and structures of these 2 metabolites are not clear. Although the biological activities of these metabolites are not determined, their contribution to clinical activity of pamiparib should be marginal because of their limited exposure in human plasma.

The M3 metabolite was also the most abundant metabolite in human urine and feces. M3 was also identified as a metabolite in liver microsomes across species and in rat plasma, urine, and feces (data on file); therefore, M3 is not considered a metabolite unique to humans. Unchanged pamiparib was observed in the urine of all patients but was only 2.11% of the administered dose during radioanalysis, which is consistent with the low CL_R_ of pamiparib determined by an LC‐MS/MS method. Unchanged pamiparib was 1.11% (range, 0.85%‐1.35%) of the dose in feces, suggesting that absorption of pamiparib was close to completion from the gastrointestinal tract in all 4 subjects. Pamiparib is a metabolic stable compound and first‐pass effect is estimated to be low. As a result, pamiparib is expected to have a high bioavailability and low clearance, consistent with its preclinical PK profiles (data on file). The metabolite profiling of pamiparib and low CL_R_ suggests metabolism is the major clearance pathway of pamiparib in humans.

Incubation of pamiparib with recombinant P450 isozymes CYP2C8 and CYP2D6 generated M2, and incubation with CYP3A and CYP2D6 generated M1. In vitro P450 phenotyping studies of pamiparib in liver microsomes showed that the CYP2C8 inhibitor montelukast and the CYP3A inhibitor ketoconazole inhibited pamiparib metabolism in human liver microsomes, but CYP2D6 inhibitor quinidine had a minimal effect on pamiparib metabolism. The biotransformation metabolic pathway suggests M1 and M2 are likely the precursor metabolites of M3. Based on these experiments, it appears that multiple P450 enzymes, including CYP2C8 and CYP3A, could contribute to pamiparib metabolism. The effect on pamiparib inflicted by coadministered drugs that inhibit or induce these enzymes needs to be further assessed.

In summary, pamiparib was well absorbed and extensively metabolized after a single 60‐mg dose administered orally. Urinary excretion represented the predominant route of elimination of pamiparib‐related drug material with minimum renal clearance of unchanged parent. Metabolism via oxidation, likely by CYP2C8, and CYP3A, is responsible for clearing pamiparib from systemic circulation. In this study, a single 60‐mg oral dose of [^14^C]‐pamiparib was well tolerated in patients with advanced and/or metastatic solid tumors.

## Conflicts of Interest

S.M., C.A.‐V., H.Z., Z.T., D.S., and S.S. are employees of BeiGene, Ltd., with stock options. D.P. reports grants and personal fees from B.M.S., AstraZeneca, Nucana Inc., Bayer, Sirtex, Roche, and Esai. R.F. has nothing to disclose.

## Funding

BeiGene, Ltd., provided financial support for this article, including writing and editorial assistance by Amit Lugade, PhD, Regina Switzer, PhD, and Elizabeth Hermans, PhD (OPEN Health Medical Communications, Chicago, Illinois). Professional medical writers, funded by BeiGene, Ltd., assisted with the development and submission of this article under the authors’ guidance.

## Author Contributions

Participated in research design: S.M., Z.T., C.A.‐V., S.S. Conducted experiments: S.M., Z.T., C.A.‐V., H.Z., D.S., D.P., R.F. Contributed new reagents or analytic tools: S.M., Z.T., D.S. Performed data analysis: S.M., H.Z., Z.T., C.A.‐V., S.S. Wrote or contributed to the writing of the manuscript: H.Z., S.M., Z.T., C.A.‐V., S.S., D.S., D.P., R.F.

## Data‐Sharing Statement

On request and subject to certain criteria, conditions, and exceptions, BeiGene will provide access to individual deidentified participant data from BeiGene‐sponsored global interventional clinical studies conducted for medicines (1) for indications that have been approved or (2) in programs that have been terminated. BeiGene will also consider requests for the protocol, data dictionary, and statistical analysis plan. Data requests may be submitted to medicalinformation@beigene.com.

## Supporting information

Supplementary information
