# Supplementary material for: Human Mass Balance and Metabolite Profiling of [14C]‐Pamiparib, a Poly (ADP‐Ribose) Polymerase Inhibitor, in Patients With Advanced Cancer
Source: Clin Pharmacol Drug Dev. 2021 Apr 19;10(9):1108–20. doi: 10.1002/cpdd.943 (PMC8453745; doi:10.1002/cpdd.943)
Supplement: Supplementary file 1 — Supplementary information [file CPDD-10-1108-s001.docx]

**Table S1**. Percent of Radioactive Dose as [^14^C]‑Pamiparib or Metabolites in Pooled Urine and Feces Samples after a Single Oral Dose of [^14^C]-Pamiparib to Patients (60 mg, 100 µCi)

| **Component** | **Retention** | **Proposed** | **% of Radioactive Dose** | |
| --- | --- | --- | --- | --- |
| **Designation** | **Time (Minutes)** | **Identification** | **Urine** | **Feces** |
| **M12** | 2.83-3.00 | unknown |  | 0.265 |
| **M13** | 4.67 | unknown |  | 0.13 |
| **M14** | 6.83-7.00 | unknown | 0.384 |  |
| **M15** | 15.83-17.17 | unknown |  | 0.763 |
| **M16** | 18 | unknown | 0.187 |  |
| **M17** | 18.67-20.33 | unknown | 0.646 | 0.078 |
| **M18** | 19.33-19.67 | unknown |  | 0.0897 |
| **M20** | 20.50-21.00 | unknown |  | 0.473 |
| **M8** | 21.67-23.17 | oxy-glucuronide | 2.15 |  |
| **M22** | 23.83 | unknown |  | 0.0574 |
| **M23** | 23.50-24.33 | unknown |  | 0.247 |
| **M24** | 24.67 | unknown |  | 0.0803 |
| **M25^a^** | 24.00-25.33 | di-oxidized pamiparib | 8.05 | 1.3 |
| **M10^a^** |  | pamiparib hydrate |  |  |
| **Pamiparib** | 29.50-32.00 | BGB-290 (parent) compound) | 2.11 | 1.11 |
| **M1** | 31.50-32.83 | BGB-4033 | 0.53 |  |
| **M27** | 34.17-34.33 | unknown |  | 0.154 |
| **M30** | 35.67 | unknown |  | 0.0504 |
| **M31** | 38.67-39.33 | unknown |  | 0.117 |
| **M32** | 40.17 | unknown |  | 0.0594 |
| **M7** | 41.17-42.83 | di-oxidized pamiparib | 3.1 | 1.24 |
| **M4** | 41.50-43.33 | di-oxy-dehydro-pamiparib | 3.75 | 0.9 |
| **M33** | 42.50-44.17 | di-oxy-dehydro-pamiparib | 1.08 | 0.157 |
| **M3** | 47.33-49.17 | oxy-dehydro-pamiparib | 28.5 | 8.71 |
| **M5** | 49.50-50.17 | oxy-dehydro-pamiparib | 1.97 | 1.32 |
| **M34** | 50.5 | unknown |  | 0.153 |
| **M35** | 52.83 | unknown |  | 0.0808 |
| ^a^ M25 and M10 coeluted. | | | | |
